# Autologous BMAC Therapy Improves Spinal Degenerative Joint Disease in Lower Back Pain Patients

**DOI:** 10.3389/fmed.2021.622573

**Published:** 2021-03-18

**Authors:** Abed El-Hakim El-Kadiry, Carlos Lumbao, Moutih Rafei, Riam Shammaa

**Affiliations:** ^1^Laboratory of Thrombosis and Hemostasis, Research Center, Montreal Heart Institute, Montreal, QC, Canada; ^2^Department of Biomedical Sciences, Université de Montréal, Montreal, QC, Canada; ^3^Canadian Centre for Regenerative Therapy, Toronto, ON, Canada; ^4^Department of Pharmacology and Physiology, Université de Montréal, Montreal, QC, Canada; ^5^Department of Microbiology, Infectious Diseases, and Immunology, Université de Montréal, Montreal, QC, Canada; ^6^Molecular Biology Program, Université de Montréal, Montreal, QC, Canada; ^7^Department of Microbiology and Immunology, McGill University, Montreal, QC, Canada; ^8^Department of Family and Community Medicine, University of Toronto, Toronto, ON, Canada

**Keywords:** spine, facet joints, lower back pain, intervertebral discs, bone marrow aspirate concentrate, magnetic resonance imaging

## Abstract

Spinal degenerative joint disease (DJD) is associated with lower back pain (LBP) arising from the degeneration of intervertebral discs (IVD), facet joints, intertransversarii muscles, and interspinous ligaments among other anatomical structures. To circumvent the socioeconomic burdens and often-problematic surgical options imposed by DJD therapy, cell-based biologic modalities like bone marrow aspirate concentrate (BMAC) have been investigated in pre-clinical and clinical settings, mostly for IVD degeneration (IDD), with encouraging outcomes. In this study, we evaluated the differences in therapeutic benefits of BMAC between IVD- and facet joint-originating chronic LBP. Eighteen patients diagnosed with chronic LBP met the selection criteria. Following discography and provocation testing, 13 patients tested positive and were assigned into IDD-associated LBP (1st arm), while the remaining 5 tested negative and were assigned into facetogenic LBP (2nd arm). Autologous BMAC was injected intradiscally in the 1st arm, while the 2nd arm received posterior spinal chain injections. No procedure-related serious events ensued. Clinical improvement was evaluated over 12 months based on pain and functionality questionnaires (VAS, BPI, RAND-36), opioid use, and changes in disc parameters assessed by magnetic resonance imaging (MRI). Ameliorated VAS and BPI scores differed significantly between both arms in favor of IDD patients who also took significantly less opioids. Average RAND-36 scores showed no significant difference between groups albeit a trend suggesting improvement was observed in IDD patients. MRI scans conducted on IDD patients demonstrated marked elevation in disc height and spinal canal space size without worsening disc quality. Overall, this is the first study investigating the potency of BMAC as an IDD treatment in Canada and the first globally for addressing facetogenic pain using cellular therapy.

**Graphical Abstract:**
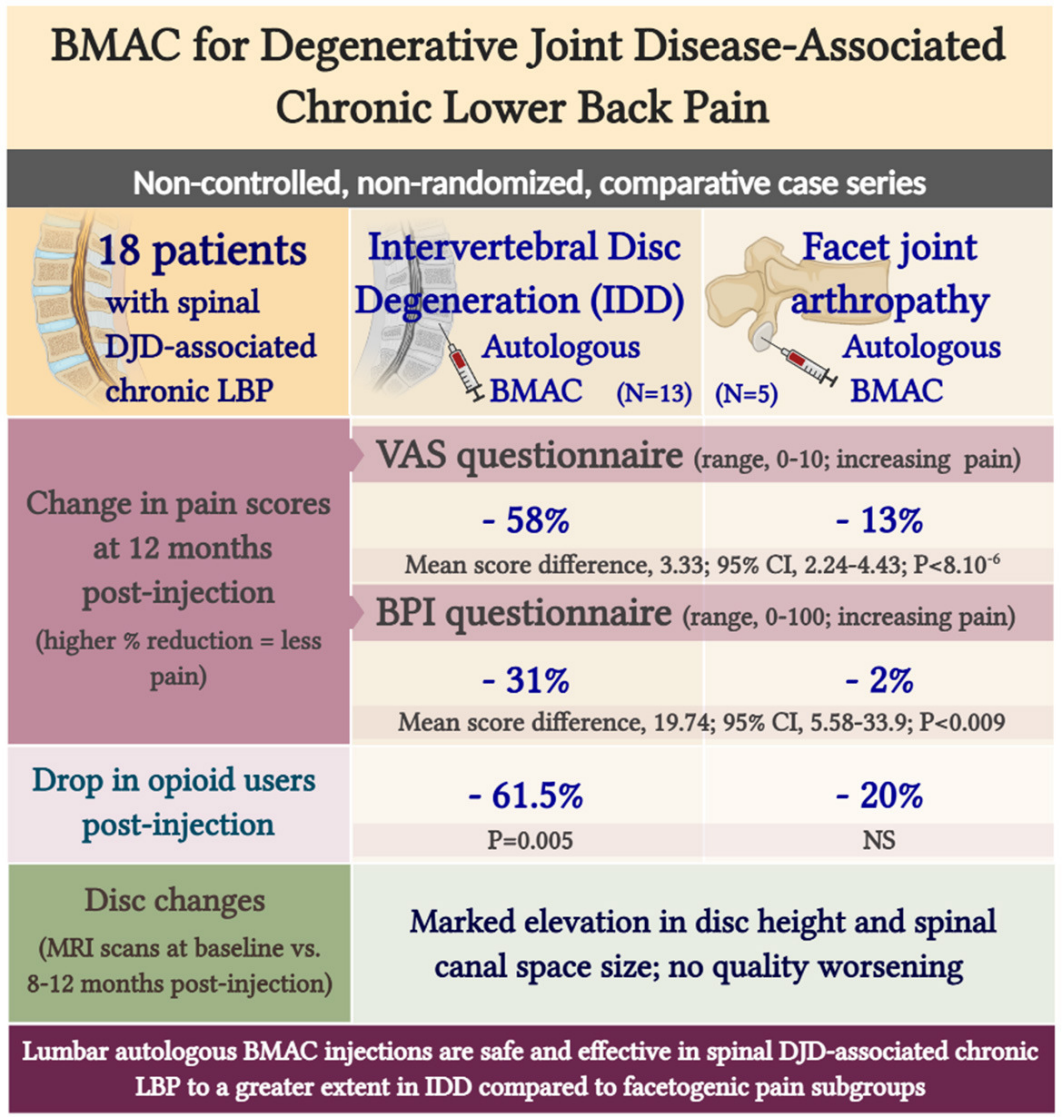
Lumbar autologous BMAC injections are safe and effective in spinal DJD-associated chronic LBP to a greater extent in IDD compared to facetogenic pain subgroups.

## Introduction

Lower back pain is a commonly confounding and costly health problem (1–4). In Canada, at least 84% of adults experience at least 1 episode of LBP during their lifetime (5–7). Behind LBP are non-anatomical and anatomical factors such as herniated discs, muscular strain, ligament strain, facet joint-mediated pain, and degenerative disc disease (DDD) (8, 9). Although non-invasive imaging techniques cannot localize the true source of LBP (10, 11), anatomical LBP associated with DJD remains provocatively diagnosed by discography despite all controversy (10).

The standard of care for chronic LBP includes exercise, heat/cold therapy, pharmacological treatments, and/or surgery. However, anatomical LBP associated with DJD remains difficult to treat since current therapeutic regimens do not address disc regeneration, but are rather limited to symptomatic relief and/or improving spinal range of motion (ROM) to preserve patient functionality (12). Biological modalities, such as stem cell transplantation, have been exploited in IDD with the aim of promoting disc healing and preserving spinal kinematics with minimal invasiveness (13, 14). Over the past decade, mesenchymal stromal cells (MSCs) have secured considerable attention in cell-based research due to their distinguished plasticity, multi-lineage differentiation potential, and secretome-mediated effects (15, 16). Likewise, bone marrow aspirate concentrate (BMAC), which consists of multiple stem cell fractions including MSCs, carries a therapeutic promise in knee DJD (17) and spinal DJD (14, 18). In knee DJD, BMAC/MSCs induce significant macroscopic, histopathological, and radiological changes by 6–20 weeks in animal models and improve pain and functionality of patients without severe adverse events (19). In spinal DJD, BMAC/MSCs similarly show safety in patients and are effective in relieving pain and ameliorating function for 12 and up to 36 months after injection (20–22). In MRI, these clinical improvements confound with an increase in the water content of treated IVDs in two studies (23). Nevertheless, current clinical evidence remains of low quality due to the paucity of high-level randomized, controlled trials; the unstandardized treatment preparation and dosing; the differences in patients' baseline disease grade; and the variability in follow-up measurements (18, 19). Most spinal DJD studies have also sought to target IVD with MSC/BMAC injections, leaving other spinal structures involved in DJD like facet joints, intertransversarii muscles, and interspinous ligaments out of scope (24).

We herein report the first Canadian retrospective, non-randomized, comparative case series with the goal to evaluate differences in pain and functionality outcomes in DJD-associated LBP between IDD and facetogenic pain patients in response to a single lumbar autologous BMAC injection. Outcomes were assessed by measuring: (i) longitudinal changes in pain and functionality using the clinically validated pain scoring systems, the visual analog scale pain score (VAS) (25), the Back Pain Index (BPI) (26–28), and the RAND-36 survey on mental and physical functioning (29), (ii) changes in frequency of opioid users, and (iii) disc recovery using multiple parameters tracked by MRI scans.

## Materials and Methods

### Study Design

Eighteen patients with anatomical LBP and meeting the selection criteria (Table 1) presented to interventional pain practice after being referred by their primary care physicians. Diagnosis was confirmed based on history, physical examination, and diagnostic imaging (X-rays and MRIs). Screening for IDD-associated LBP was performed using provocation testing and discography. Accordingly, 13 patients testing positive on disc provocation (partial or concordant pain) were assigned to the first treatment arm. The remaining 5 patients tested negative on disc provocation (discordant pain) and were thus assigned to a second treatment arm. Before treatment, patients acknowledged the study purpose, the associated risks and benefits, and the treatment alternatives. In agreement with the local legislative and procedural institutional obligations regarding the retrospective nature of the study, ethical review and approval was not required. Informed consent forms as a written signed expression of patient voluntary participation were obtained. Patients also consented to the anonymous publishing of any collected data and to epidurals and medial branch blocks for retrospective comparative studies.

**Table 1:** Selection criteria.

**Inclusion criteria**	**Exclusion criteria**
Male or female	Active oral anticoagulants or heparin therapy
18 years of age or older	Pregnancy
Refractory low back pain persisting for ≥ 6 months	Active cancer
Disc disease Grade II or more on Pfirrmann grading on T2 MRI with or without same level facet arthropathy	Systemic infection or skin infection over the puncture site
Willingness to voluntarily participate	Allergy to contrast agent
	Solid bone fusion preventing access to the disc
	Extrusions or sequestered disc fragments
	Previous spinal surgery
	Spondylolysis
	Spondylolisthesis: ≥ grade III

### Provocation Testing and Discography

Unless non-conforming to the pre-discography inclusion criteria included in Table 1, patients were positioned on a fluoroscopy table for intradiscal access after receiving 1 g of Cefazolin intravenously in 250 ml of normal 0.9% saline over 60 min. The target location was prepared with a sterile technique using drapes and chlorhexidine (three times). At a roughly 45° cranial tilt of the C-arm, the targeted location was aligned and properly visualized. Skin was anesthetized with a 2 cc wheal of 2% lidocaine. In the same puncture location of the wheal, an 18-gauge 3.5-inch spinal needle was then inserted through the musculature, placed with fluoroscopic guidance *via* a right paramedian approach, and ultimately positioned slightly lateral to the superior articulating process (SAP) at a midpoint between the endplates. When the needle was correctly positioned, the stylet was removed to allow the insertion of a 22-gauge 7-inch spinal needle through. The needle was passed lateral to the SAP and medial to the exiting nerve root. During its passage through the annulus, resistance and back pain were noted. When pain in the extremities was reported, the needle was redirected due to potential nerve root contact. Upon disc penetration, antero-posterior and lateral disc images were obtained, both of which visualized the needle tip in the center of the disc space. The procedure was repeated at all concerned levels. Afterwards, pain was localized through the injection of a pressurizing contrast (Omnipaque 300, 0.1 cc) and subsequently assessed with a disc pressure-measuring manometer connected to the needle. The amount of pain provoked in the disc tested and while the patient was blinded was delineated as such: P0 (no pain on injection), P1 (partial concordant pain), P2 (discordant pain), and P3 (concordant pain). Discs were considered positive if P1 or P3 was recorded and concorded with the MRI. Negative levels (P2) were used as control. Simultaneously, disc quality was interpreted further by discogram findings under fluoroscopic guidance. Positive imaging findings were not considered significant if teamed with P0 levels. The patterns of imaging findings based on discography and their corresponding significance are described in Supplementary Table 1.

### BMAC Preparations

Under sterile conditions, autologous BMAC was prepared as previously described (30). Briefly, the Posterior Superior Iliac Spine was marked with ultrasound guidance for bone marrow aspiration, and 2% lidocaine was injected into the soft tissue and periosteum. An entry point was created with the introducer 14G trocar needle with which the bone was then drilled through the periosteum and cortex and into the spongy bone. Subsequently, using heparinized syringes, 1 to 6 cc were aspirated per level while slowly withdrawing until ~60 cc of BMAC were collected. BMAC was further enriched using Chondrostem, a customized and validated cell filtration device (CCRT, Toronto, Canada) for the mononuclear fraction containing MSCs (CD45^−^CD44^+^CD90^+^CD105^+^) among others (30).

### Injection Protocols

In the first treatment arm, positive discs of patients were injected with autologous 1–6 cc of BMAC into the nucleus pulposus, while negative discs were sealed with only 0.1 cc BMAC. The volume of BMAC injected per disc was determined based on the intradiscal pressure created during injection and the ability of the disc to accept the maximum volume injected at a sustained pressure between 51 and 90 psi (346.2–620.5 Kpa) (31). In the second treatment arm, patients were treated with posterior spinal chain injections of autologous 1–5 cc of BMAC per structure per level, specifically into the zygapophyseal joints (also called facet joints), multifidi, intertransversarii muscles, interspinous ligaments, and Sacroiliac joints (including intraosseous and posterior sacroiliac ligaments). The treatment modalities for both cohorts are further detailed in Supplementary Tables 2, 3.

### Outcome Evaluations and Follow-Up Assessments

The first evaluation post-procedure occurred after 2 weeks at the Canadian Center for Regenerative Therapy (CCRT, Toronto, ON, Canada). Subsequently, all patients were instructed to follow up 1, 3, 6, 9, and 12 months following injection. At each visit, range of motion (ROM) and tenderness to palpation at the joint line level were assessed. Pain and patient functionality were re-assessed as well using the clinically validated scoring systems (VAS, BPI, and RAND-36). Radiological follow-up entailed a second MRI between 8–12 months post-procedure. MRI scans were performed at different centers, and images reflecting the best anatomical integrity were chosen to assess changes in disc quality compared with the pre-procedure MRI in terms of disc height; spinal canal space size; and disc quality according to Pfirrmann grading. Specifically, disc height and spinal canal space size were measured by 2 blinded investigators, and disc quality was graded using the clinically accepted 5-level Pfirrmann scale for disc degeneration with level 5 being the utmost degenerate (32).

### Statistical Methods

Statistical analyses were conducted using SPSS 20.0 (IBM Corp., Armonk, NY). Results are depicted as mean ± standard deviation (SD) unless otherwise specified. The Shapiro-Wilk test was used to verify the normal data distribution. Independent samples *t*-test for normally and Chi-Square-test for non-normally distributed data were performed to analyze score outcome differences between baseline findings of both treatment arms. The repeated-measures general linear model (GLM) with Sidak test for pairwise comparisons was performed to investigate the influence of the treatment on the evolution of VAS, BPI, and RAND-36 scores within a group. The repeated-measures GLM with Sidak test for pairwise comparisons was used to compare change in VAS, BPI, and RAND-36 scores between treatment groups over time. As such, time was considered a within-subject variable and treatment a between-subject factor. The primary variable of interest between both groups was the effect of treatment and the difference of estimated marginal means. Based on the variable type and normality or non-normality of distribution, Paired *t*-test or Chi-Squared test was performed to analyze opioid use and MRI outcomes between pre- and post-procedure. Pearson correlation (2-tailed) was performed to detect correlations between score improvement and total BMAC volume injected in both groups. The GLM with scores as dependent variable and treatment group as fixed factor was adopted to detect significant co-variate effects (total BMAC volume injected or baseline provocation test score) and interaction effects on score improvement of both groups. *P* < 0.05 was considered statistically significant.

## Results

### Baseline Demographics and Clinical Characteristics

Of the 18 patients recruited for this study, 13 tested positive for disc provocation and were assigned into the first treatment arm (disc provocation-positive; IDD pain group). The remaining 5 patients tested negative and were assigned to the second treatment arm (disc provocation-negative; facetogenic pain group). Although no serious complications or adverse events were recorded, the increased pain reported in some patients 48 h following the procedure resolved within 5–7 days. Overall, no significant differences were detected between both groups with respect to demographics and baseline self-reported test scores obtained prior to discography (Table 2).

**Table 2:** Baseline demographics and clinical characteristics.

**Patient characteristics**	**Disc provocation-positive patients**	**Disc provocation-negative patients**	***P*-value**
Gender (*n*)	Female (6)	Female (3)	1.0^a^
	Male (7)	Male (2)	
Age, median (min-max)	63 (33–78)	57 (40–77)	0.891^b^
On Percocet (1–3 tab. p.r.n), *n* (%)	10 (76.9%)	3 (60%)	0.583^a^
VAS, mean (SD)	6 (1.87)	8 (1.58)	0.051^b^
BPI, mean (SD)	48.62 (14.71)	60.8 (13.23)	0.126^b^
SF-12, mean (SD)	56.15 (24.03)	42.8 (23.91)	0.306^b^
Pain provocation test score per disc, n (%)	P1, 9 (40.91%)	P2, 5 (100%)	
	P3, 13 (59.09%)		
Disc quality, n (%)	2, 2 (9.09%)	1, 5 (100%)	
	3, 5 (22.73%)		
	4, 7 (31.82%)		
	5, 8 (36.36%)		

a *Chi-Square-test (2-sided)*.

b *Independent samples t-test (2-tailed)*.

### BMAC Administration Improves Pain Intensity in Disc Provocation-Positive Patients

**Figure 1:**
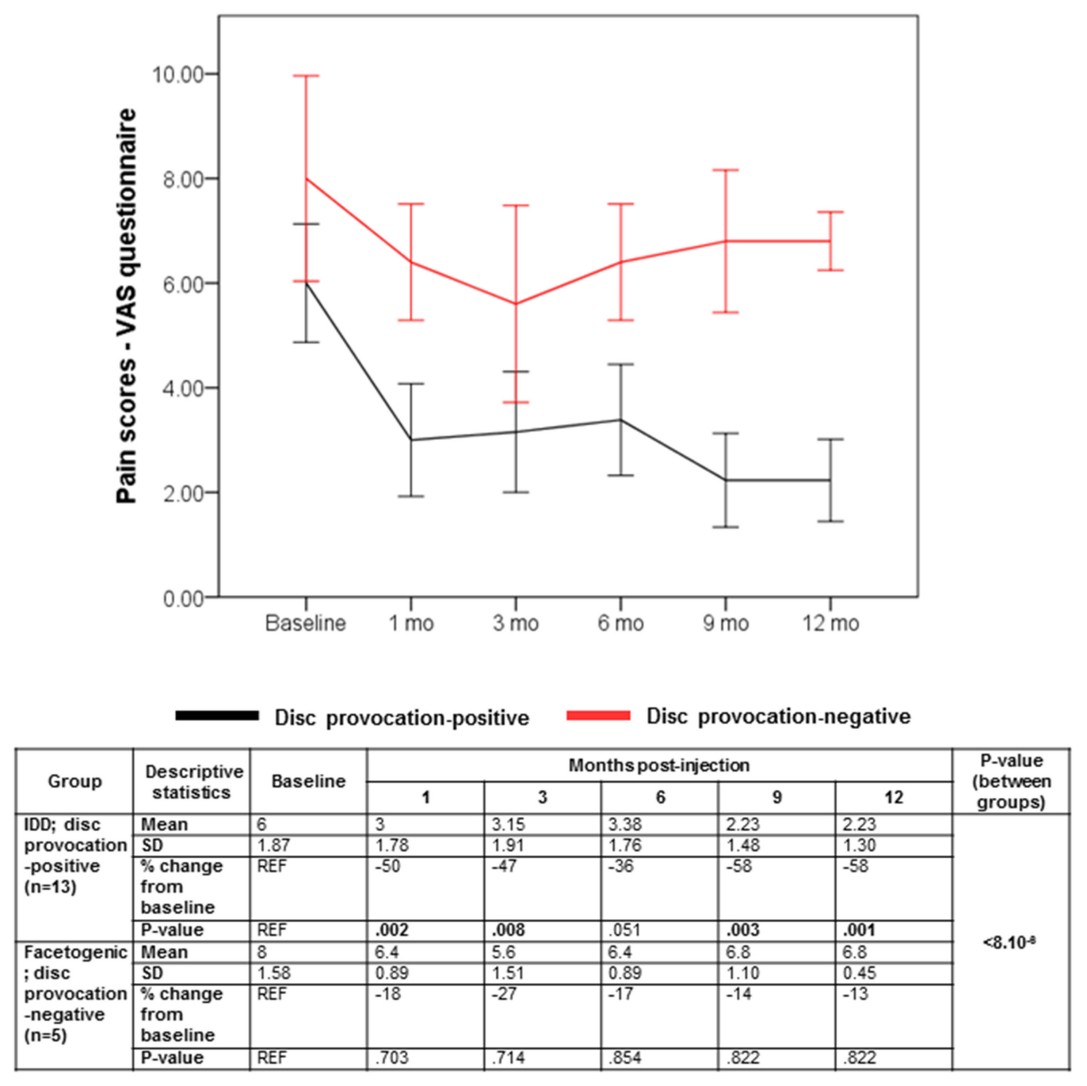
Evolution of VAS scores following treatments. Error bars are represented by 95% Confidence Interval. The repeated-measures general linear model with Sidak-test was used to calculate *p*-values of within- and between-group differences.

**Figure 2:**
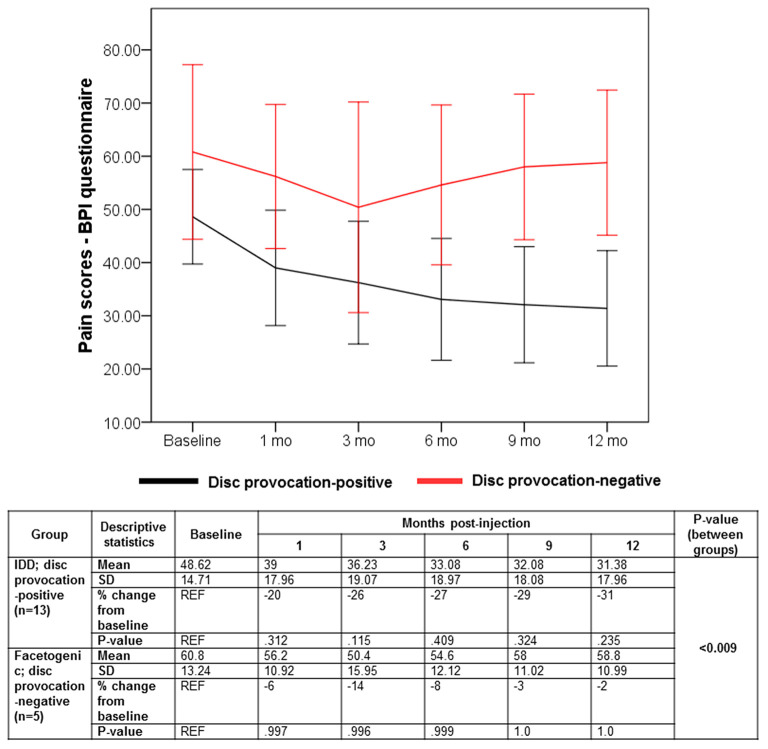
Evolution of BPI scores following treatments. Error bars are represented by 95% Confidence Interval. The repeated-measures general linear model with Sidak-test was used to calculate *p*-values of within- and between-group differences.

**Figure 3:**
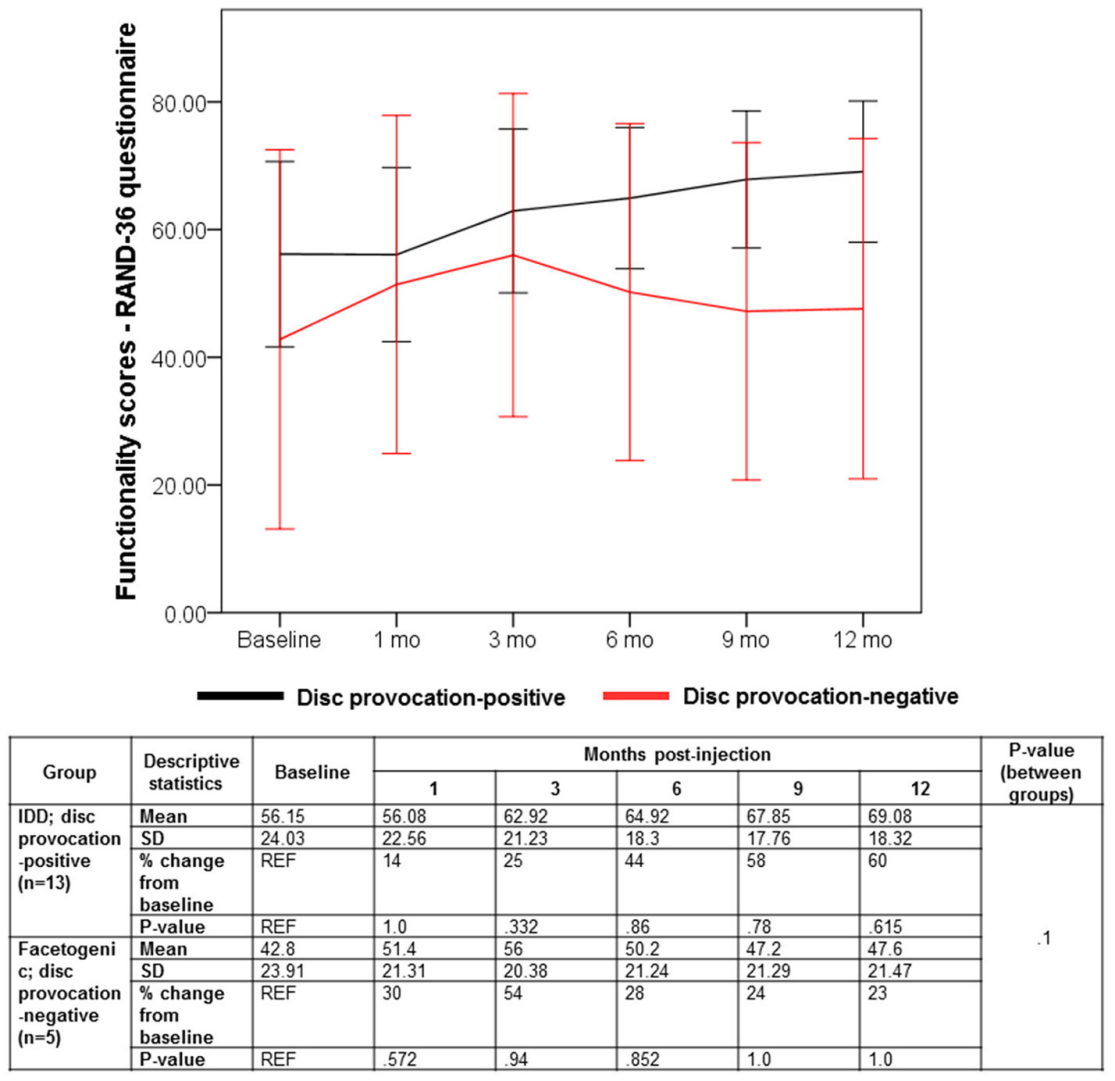
Evolution of RAND-36 scores following treatments. Error bars are represented by 95% Confidence Interval. The repeated-measures general linear model with Sidak-test was used to calculate *p*-values of within- and between-group differences.

Despite the multifaceted nature of pain, VAS is a reliable test for evaluating pain intensity variations on a scale of 0–10 cm (33, 34). Average baseline VAS scores were 6.00 (±1.87) and 8 (±1.58) in disc provocation-positive and -negative groups, respectively. Post-procedure, VAS scores differed significantly over time between both groups (*p* < 8 × 10^−6^), such that disc provocation-positive patients self-reported on average a pain score of 2.23 (±1.3) at the final follow-up (58% improvement; *p* = 0.001), while disc provocation-negative patients scored 6.8 (±0.44) (13% improvement; *p* = 0.8) (Figure 1). Patients were further evaluated using the BPI questionnaire, which provides insight into the impact of pain intensity on daily tasking (28). Average BPI at baseline was 48.62 (±14.71) in disc provocation-positive group and 60.8 (±13.24) in disc provocation-negative group. Throughout the study, improvement in BPI scores differed significantly between both groups (p < 0.009), such that at the final follow-up, disc provocation-positive patients self-reported on average a pain score of 31.38 (±17.96) (31% improvement; *p* = 0.2), while disc provocation-negative patients reported 58.8 (±10.99) (2% improvement) (Figure 2). Besides, RAND-36 was undertaken to follow up on patients' self-reported quality of life (29). Average baseline RAND-36 scores were 56.15 (±24.03) and 42.8 (±23.91) in disc provocation-positive and -negative groups, respectively. At the final follow-up, disc provocation-positive patients self-reported 69.08 (±18.32) on average in the survey (60% improvement; *p* = 0.6), while disc provocation-negative patients reported 47.6 (±21.47) (23% improvement; *p* = 1.0). Although RAND-36-based changes were not significantly different between both cohorts, an improvement trend was observed in the disc provocation-positive group (Figure 3).

### Opioid Use to Relieve Pain Was Diminished in IDD Patients Under BMAC Treatment

**Figure 4:**
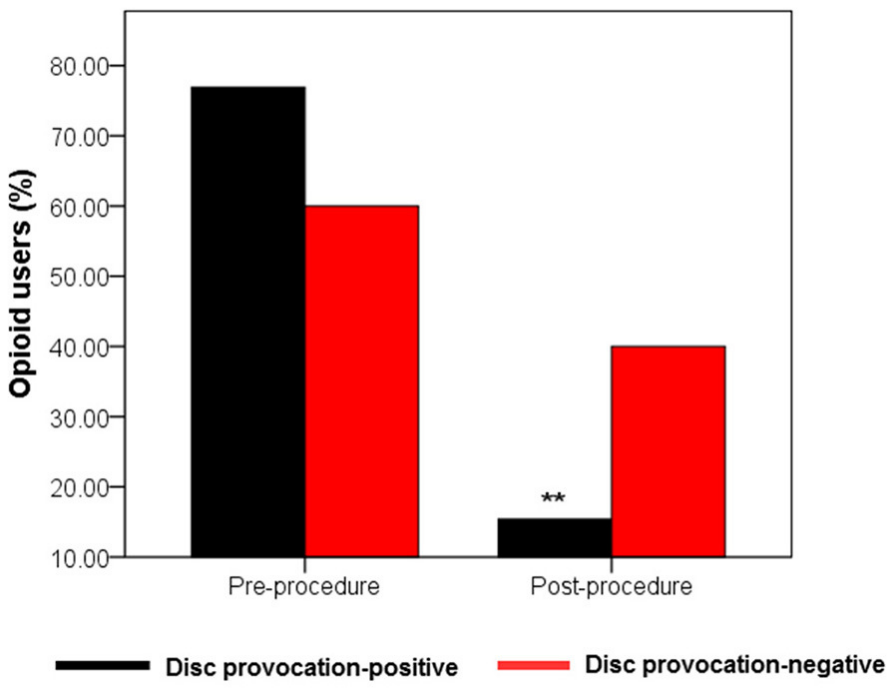
Evolution of Percocet use by treatment group. Bar graph showing percentage of Percocet users in the two treatment groups (*n*_1_ = 13; *n*_2_ = 5) between baseline and after the first month post-injection. Only disc provocation-positive patients showed a statistical difference in the frequency of Percocet users between the two time points ^**^p = 0.005 vs. pre-procedure (2-sided Chi-Square-test).

Percocet (oxycodone/acetaminophen) is an opioid analgesic indicated for the relief of moderate to moderately severe pain (35). Among IDD patients, the percentage of Percocet users markedly decreased (*p* = 0.005) from 76.9% before intervention to 15.4% after a month post-procedure. Among disc provocation-negative patients, the frequency of Percocet use decreased from 60% to 40% albeit without statistical significance (Figure 4).

### Disc Provocation-Positive Patients Exhibit Improved Disc Height and Canal Space Size and No Worsening in Disc Quality Post-BMAC Treatment

**Figure 5:**
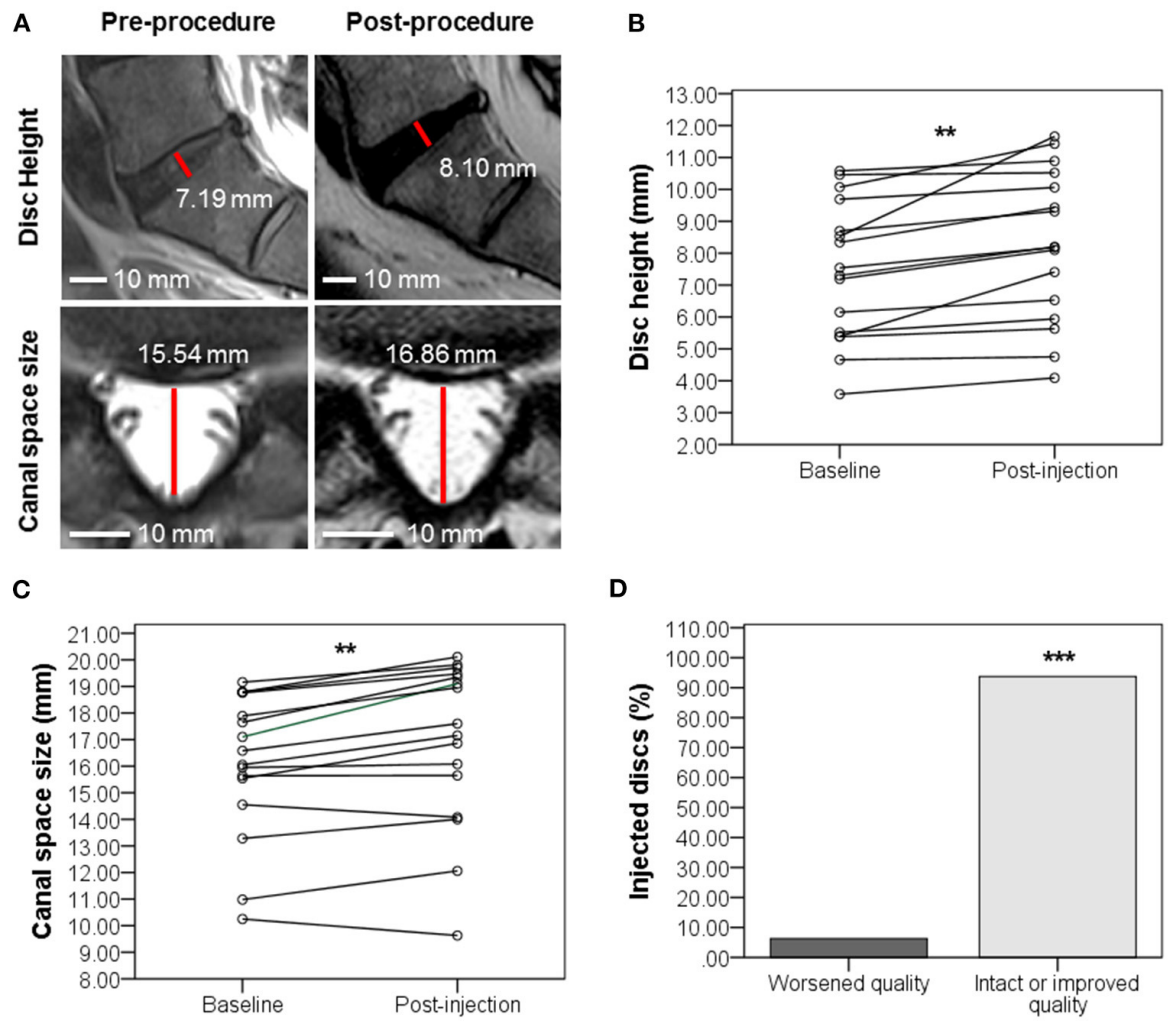
Evolution of disc quality 8–12 months post-injection in disc provocation-positive group as assessed with MRI. **(A)** Representative MRI scan showing the evolution of IVD (sagittal plane) and spinal canal space (axial view) at L5-S1 from baseline to 11 months post-injection. Disc height was elevated from 7.19 to 8.19 mm. Canal space size increased from 15.54 to 16.86 mm. **(B)** Scatter plot of disc height (mm) evolution with interpolation lines ^**^p = 0.001 (Paired *t-*test of means, *n* = 16 discs of 9 patients). **(C)** Scatter plot of canal space (mm) evolution with interpolation lines ^**^p = 0.001 (Paired *t-*test of means, *n* = 16 discs of 9 patients). **(D)** Bar graph showing the percentage of disc changes after injection. ^***^P = 0.001 (Chi-Square test, *n* = 16 discs of 9 patients).

Eight to 12 months post-injection, 9 of 13 disc provocation-positive patients were followed up with MRI to anatomically corroborate their improvement in self-reported pain and functionality. Sixteen total discs were imaged and interpreted for changes in three parameters. Figure 5A shows MRI scans of one patient with disc parameter changes representative of the cohort mean. MRI scans of the remaining patients are provided in the supplementary material (Supplementary Figures 1–6). Indeed, on average, disc height significantly increased (*p* = 0.001) by 11.45% from 7.44 mm (±2.18 mm) at baseline to 8.26 mm (±2.38 mm) post-treatment (Figure 5B). Canal space size significantly increased (*p* = 0.001) by 4.66% on average from 16.06 mm (±2.7 mm) at baseline to 16.85 mm (±3.09 mm) post-treatment (Figure 5C). Discs whose quality according to Pfirrmann grading did not worsen were significantly more numerous by 87.5% than discs that worsened following injection. Indeed, only 1 disc (belonging to 1 patient) worsened by 1 grade (from 2 to 3), whereas 4 discs (belonging to 4 patients) improved by 1–2 grades. The remaining 11 discs (belonging to 7 patients) partook no grade change (Figure 5D).

## Discussion

Current standard of care for facet joint- and IDD-associated chronic LBP manages pain and functionality without eliciting a long-term effect. Although more invasive approaches such as surgery, decompression, radiofrequency or intra-articular corticosteroid injection yield transient outcomes, they remain associated to certain complications and financial burdens (36, 37). Biological modalities like stem cell therapy, on the other hand, were shown to harbor a regenerative potential in several pre-clinical (13, 14) and clinical studies (18, 23, 38). Although BMAC administration was demonstrated to be clinically safe and promising for several orthopedic conditions including IDD (18, 21, 30, 39), no studies addressed or supported its use for facetogenic chronic LBP. In fact, all studies targeting facet joint-mediated pain were conducted using platelet rich plasma or prolotherapy (24). As such, we evaluated in this study the therapeutic effect of lumbar injection using autologous BMAC (1–5 ml per structure per level) in patients with chronic LBP. Besides the absence of adverse events, improved lower back pain was observed and corroborated by MRI scans for disc quality.

In terms of pain intensity, 12 of 13 discogenic patients reported a significant 20–100% improvement (58% on average) in VAS scores with most patients reporting a non-significant 7–94% improvement (31% on average) in BPI scores between baseline and 12 months post-injection. The patients who showed no overall pain amelioration in both tests were either the eldest (patient 4) or had the highest number of positive discs and/or worst disc quality (patients 5 and 13). Between both treatment groups, average VAS and BPI scores differed significantly in favor of IDD patients. Similarly, IDD patients experienced a statistically significant 61.5% drop in opioid use.

In terms of functionality, RAND-36 scores improved, albeit non-significantly, by an average of 60% in 10 of 13 IDD patients with 8 of 13 patients reporting better scores in all three tests. With respect to facetogenic pain patients, RAND-36 scores improved non-significantly by 23% on average and in 3 of 5 patients. Between the remaining 2 patients whose RAND-36 scores did not improve (patients 1 and 5), one did not show pain amelioration in VAS and BPI and had the highest baseline (patient 1). In addition, 1 out of 5 patients reported better scores in all three tests (patient 2).

Most IDD patients underwent MRI 8–12 months post-injection to anatomically corroborate their ameliorated pain and functionality. On average, IVDs and spinal canal spaces witnessed, respectively, a significant 11.45% increase in height (equivalent to +0.82 mm) and a marked 4.66% increase in size (equivalent to +0.79 mm). In terms of disc quality, only 6.25% of injected discs worsened, compared to 93.75% that improved or exhibited no changes. Noteworthy, despite choosing the MRI scans with the most integrity for comparison and the blinding of IVD measurements, there exists a possibility of data interference imposed by the involvement of different imaging centers and associated issues of MRI standardization (different machine qualities, software, and image slicing levels).

The therapeutic benefits of BMAC (Graphical Abstract) can be linked to their enriched content in MSCs, which are unique in their multi-lineage differentiation potential as well as anti-inflammatory and pro-angiogenic auxiliary effects (40). In investigational regenerative IDD therapy, MSCs have been shown to induce disc cell matrix proliferation in rabbits (41) and augment disc water content in humans (42, 43). It was also shown that the interaction between MSCs and IVD bestows upon MSCs an IVD-like phenotype and stimulates the disc to synthesize a new matrix for IVD repair (44, 45). Currently, a randomized, controlled, double blind trial is investigating the efficacy of intradiscally injected autologous MSCs in chronic LBP (NCT04759105). Furthermore, BMAC is rich in mononuclear cell populations including endothelial progenitor cells, platelets, and cytokines, all of which are known to promote bone regeneration (46). Of note, although most studies—ours included—corroborate the safety and benefits of BMAC/MSCs in IDD among other orthopedic conditions, the comparison of data between studies is still difficult due to multiple challenges that are yet to be overcome; these include the unstandardized preparation and dosage of injectables, the unclear mechanisms of action of the biologic, and the variable endpoints and measurement of outcomes (23).

**Figure 6:**
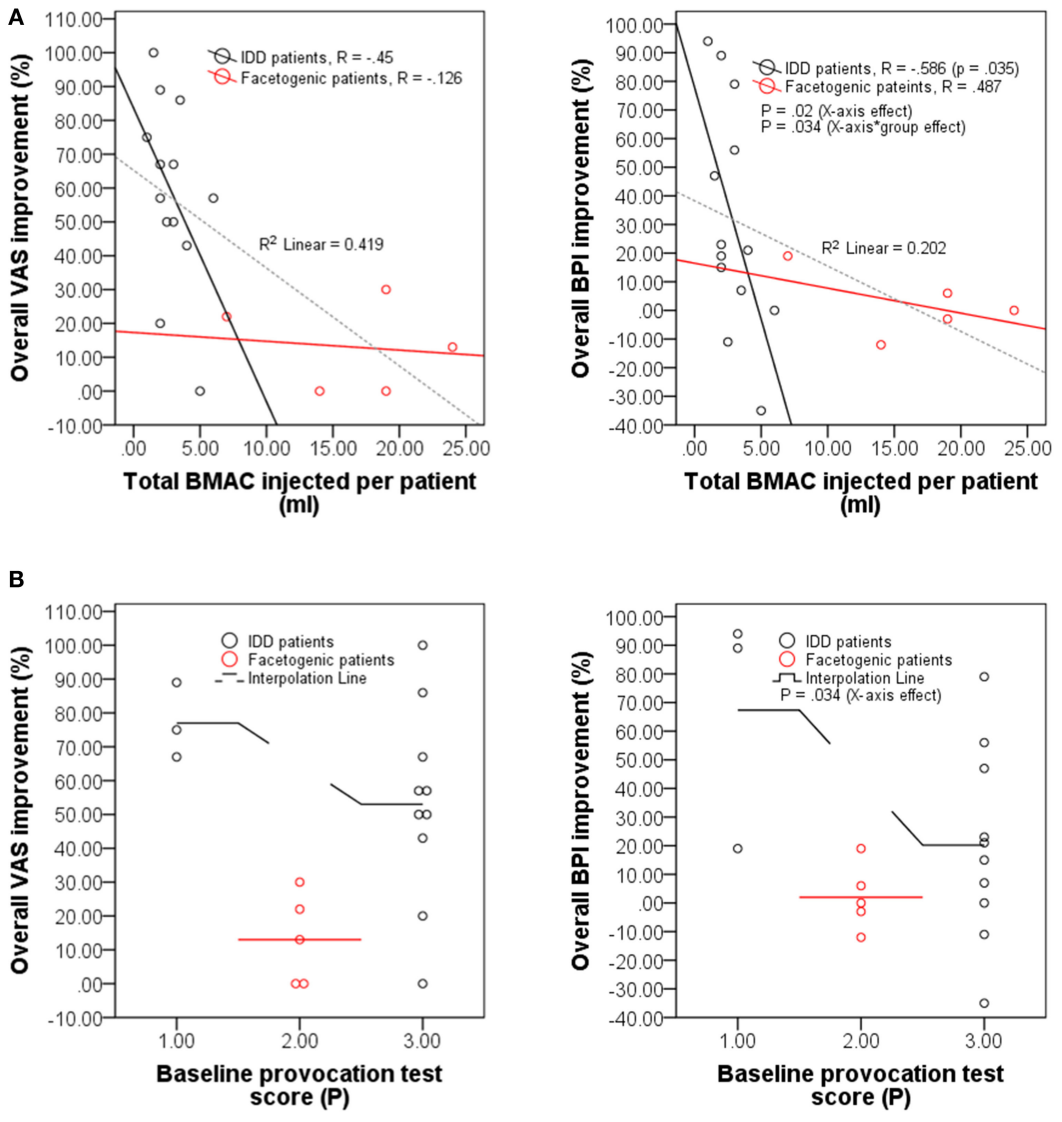
The therapeutic effect of BMAC is volume-independent. **(A)** Scatter plots displaying overall improvement (in percentage) self-reported by IDD patients (*n* = 13) vs. facetogenic patients (*n* = 5) at 12 months post-injection under VAS and BPI questionnaires as a function of total BMAC volume injected (ml) per patient. **(B)** Scatter plots with interpolation lines displaying overall improvement (in percentage) of IDD (*n* = 13) and facetogenic patients (*n* = 5) at 12 months post-injection under VAS and BPI questionnaires as a function of maximum pain score (*P*) recorded during provocation testing before treatment.

Previously, the concentration of intradiscally injected MSCs was shown to positively correlate with overall patient beneficence (21, 47). Contrastingly, our observations demonstrate a significant negative correlation between BPI (but not VAS) improvement and total BMAC volume injected in IDD patients (*R* = −0.586; *p* = 0.035; Figure 6A); a significant effect of total BMAC volume injected on BPI (but not VAS) improvement in IDD and facetogenic patients (*p* = 0.02; Figure 6A); and a significant effect of baseline pain score recorded during provocation testing on BPI (but not VAS) improvement in both groups (*p* = 0.034; Figure 6B). This indicates that the therapeutic effect of BMAC may not rely on the volume of injection but may rather depend on the site of injection or localization in damaged tissues. On the same note, these results might be further reflected by the treatment protocol of IDD patients, some of whom received different injection volumes between spine segments (Supplementary Table 2) seemingly due to disc leakage that hinders reaching the standard pressure (51–90 psi) upon injection (section Injection protocols). Therefore, to achieve and sustain that pressure, these patients required higher BMAC volumes, which might suggest improper tissue retention of the biologic and subsequent worse outcomes.

Although our results highlight improved outcomes in most treated spinal DJD patients, the study weaknesses are its: (i) small sample size, (ii) differences in group sizes, (iii) uncontrolled nature of the study, (iv) unstandardized concentration and application of the biologic, and (v) MRI standardization issues. A larger randomized controlled trial with a more robust standard operating procedure is therefore needed to authenticate the efficacy of the investigated biologic in the selective treatment of IDD-associated chronic LBP. Whether higher BMAC volumes and/or multiple injections can improve clinical outcomes and augment statistical power is also an open avenue for future investigations.

## Conclusion

Lumbar autologous BMAC injections safely and effectively reduced pain and opioid intake, ameliorated mobility, and induced parallel anatomical disc changes in spinal DJD-associated chronic LBP to a greater extent in IDD compared to facetogenic pain subgroups. These data further exhibit the clinical utility of BMAC, which may prove to be a substitute for IDD surgery following wider-scale studies.

## Data Availability Statement

The raw data supporting the conclusions of this article will be made available by the authors, without undue reservation.

## Ethics Statement

Ethical review and approval was not required for the study on human participants in accordance with the local legislation and institutional requirements. The patients/participants provided their written informed consent to participate in this study.

## Author Contributions

AE-K analyzed the data and wrote the first draft of the manuscript. CL managed the logistics of the study and participated in the treatment procedures. MR conceived the study, analyzed data, and edited the manuscript. RS conceived and conducted the studies, analyzed the data and edited the manuscript. All authors contributed to the revision of the manuscript.

## Conflict of Interest

This work was supported by the Merck Frosst Start-up funds (Grant #RH000569) provided by Université de Montréal and by a grant from Merck-Sharp and Dohme Corp. (Grant #SFMERE58) to MR. MR holds a Fonds de la Recherche en Santé du Québec Junior I and II Awards. The remaining authors declare that the research was conducted in the absence of any commercial or financial relationships that could be construed as a potential conflict of interest.

## Abbreviations

DJD: degenerative joint disease
LBP: lower back pain
IVD: intervertebral disk
BMAC: bone marrow aspirate concentrate
IDD: intervertebral disc degeneration
MRI: magnetic resonance imaging
DDD: degenerative disc disease
ROM: range of motion
MSC(s): mesenchymal stem cell(s)
BMA: bone marrow aspirate
VAS: visual analog scale
BPI: back pain index
GLM: general linear model.
