# Supplementary material for: Autologous BMAC Therapy Improves Spinal Degenerative Joint Disease in Lower Back Pain Patients
Source: Front Med (Lausanne). 2021 Mar 18;8:622573. doi: 10.3389/fmed.2021.622573 (PMC8012529; doi:10.3389/fmed.2021.622573)
Supplement: Supplementary file 1 [file Data_Sheet_1.PDF]

Supplementary Material

1 Supplementary tables

Supplementary Table S1. Discography imaging findings and their interpretation.

Discogram finding	Disc quality	Contrast distribution pattern	Interpretation
**Cotton ball**	1	Central to disc, ovoid shape	Undegenerated disc
**Lobular**	2	Centralized, two distinct arcs juxtaposed to both end plates	Mature disc, nucleus starting to grow into fibrous mass
**Irregular**	3	Extension outside the central nucleus, no annular extension	Degenerated disc, nuclear as well as inner annular fissures
**Fissured**	4	Extension into the outer annular margins	Degenerated disc, radial fissures extending into posterior annular margins
**Ruptured**	5	Contrast escapes into the epidural space	Degenerated disc (can be at any degeneration stage), complete radial fissures and annular tear
**End plate fracture**	6	Abnormal diffusion	End plate disruption

Supplementary Table S2. The single treatment modality of disc provocation-positive group.

Patient	Positive disc level(s) injected	BMAC injected per level (ml)
**1**	L5-S1	1.5
**2**	L3-L4	2
	L4-L5	1
**3**	L5-S1	2
**4**	L3-L4	3
	L4-L5	2
**5**	L4-L5	1
	L5-S1	1.5
**6**	L5-S1	2
**7**	L5-S1	3
**8**	L2-L3	2
	L4-L5	1.5
**9**	L4-L5	1
	L5-S1	1
**10**	L4-L5	1
	L5-S1	1
**11**	L4-L5	1
**12**	L3-L4	2
	L5-S1	2

**13**	L1-L2	3
	L2-L3	2
	L3-L4	1

Supplementary Table S3. The single treatment modality of disc provocation-negative group.

Patient	Facet, muscle, and ligament injected per level	BMAC injected per structure (ml)
**1**	L2-L3: facet (R)	2
	L3-L4: muscle (R)	2
	L4-L5: facet (B); muscle (L)	2 each; 2
	L5-S1: facet (B); muscle (R)	1 (L) and 4 (R); 4
**2**	L4-L5: ligament (L); facet (R)	1; 2
	L5-S1: muscle (B)	2 each
**3**	L3-L4: facet (B)	2 each
	L4-L5: facet (B)	2 (L) and 3 (R)
	L5-S1: facet (B)	3 (L) and 2 (R)
**4**	L3-L4: facet (B)	5 each
	L4-L5: facet (B)	5 each
	L5-S1: facet (B)	2 each
**5**	L3-L4: facet (B)	2 (L) and 3 (R)
	L4-L5: facet (B)	3 each
	L5-S1: facet (B); muscle (R)	3 each; 2

L, left; R, right; B, both

2 Supplementary Figures

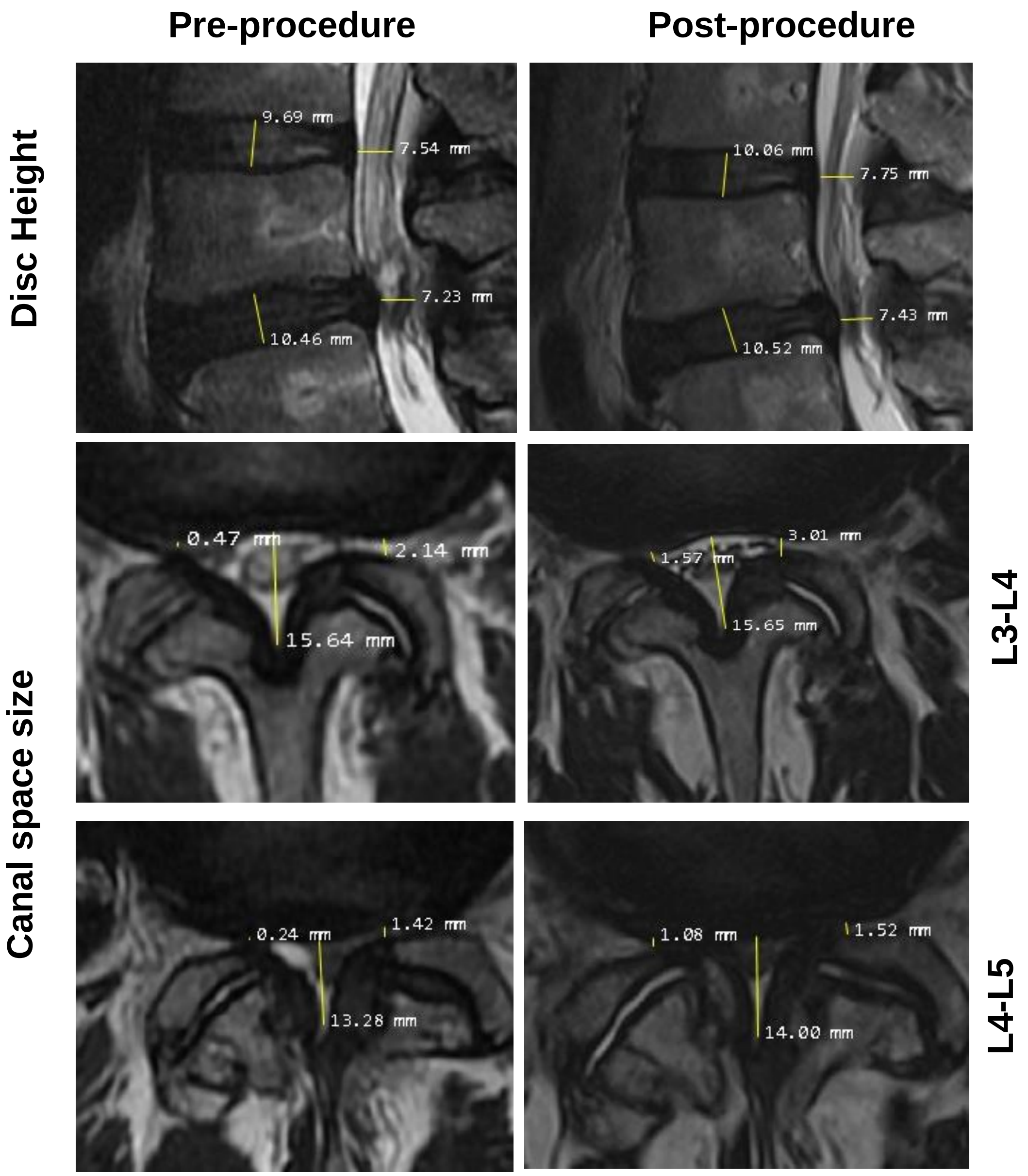

Supplementary Figure S1. MRI pictogram showing the evolution of disc height and canal space size of Patient 2.

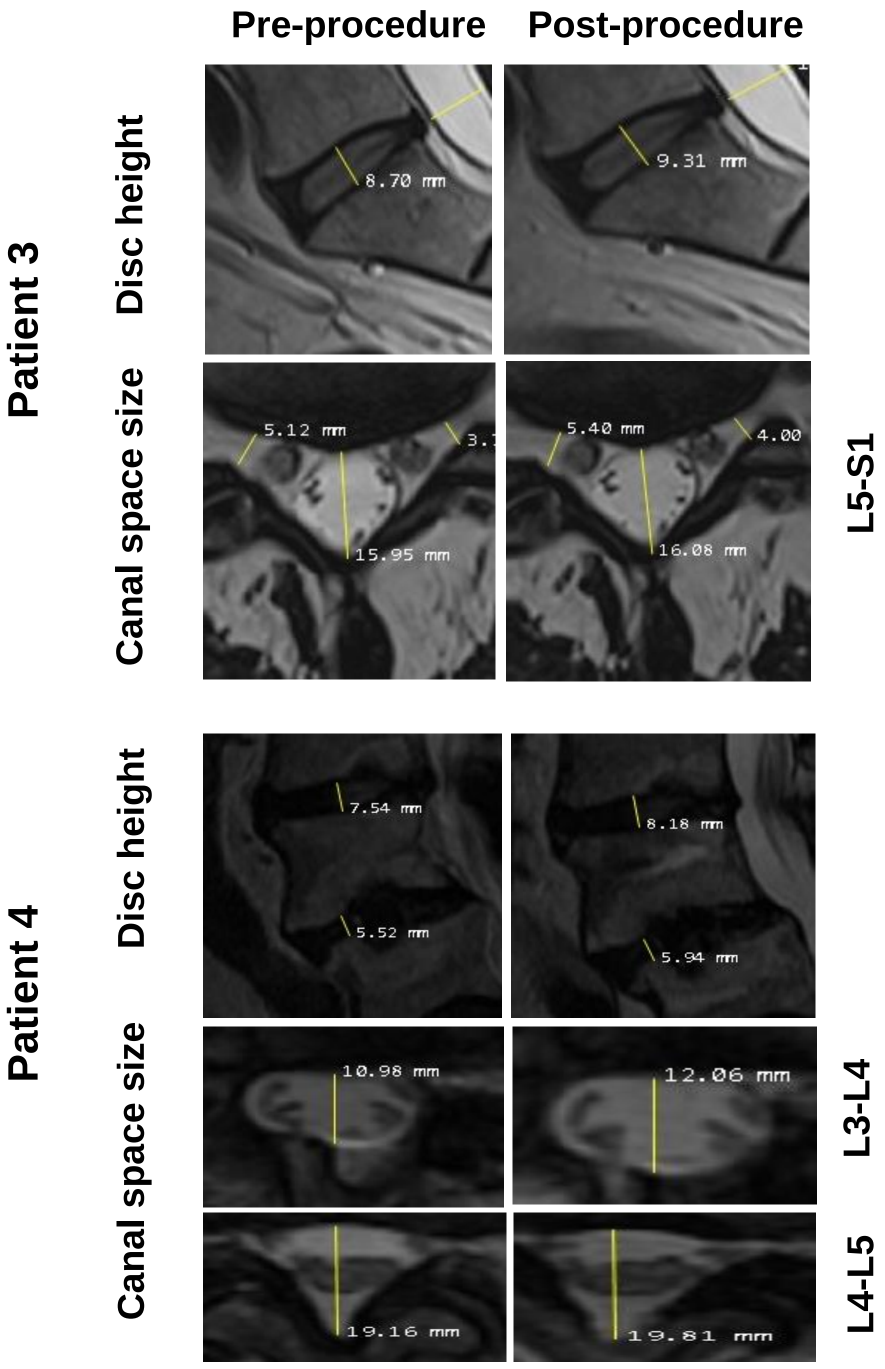

Supplementary

Figure S2. MRI pictogram showing the evolution of disc height and canal space size of Patient 3 and Patient 4.

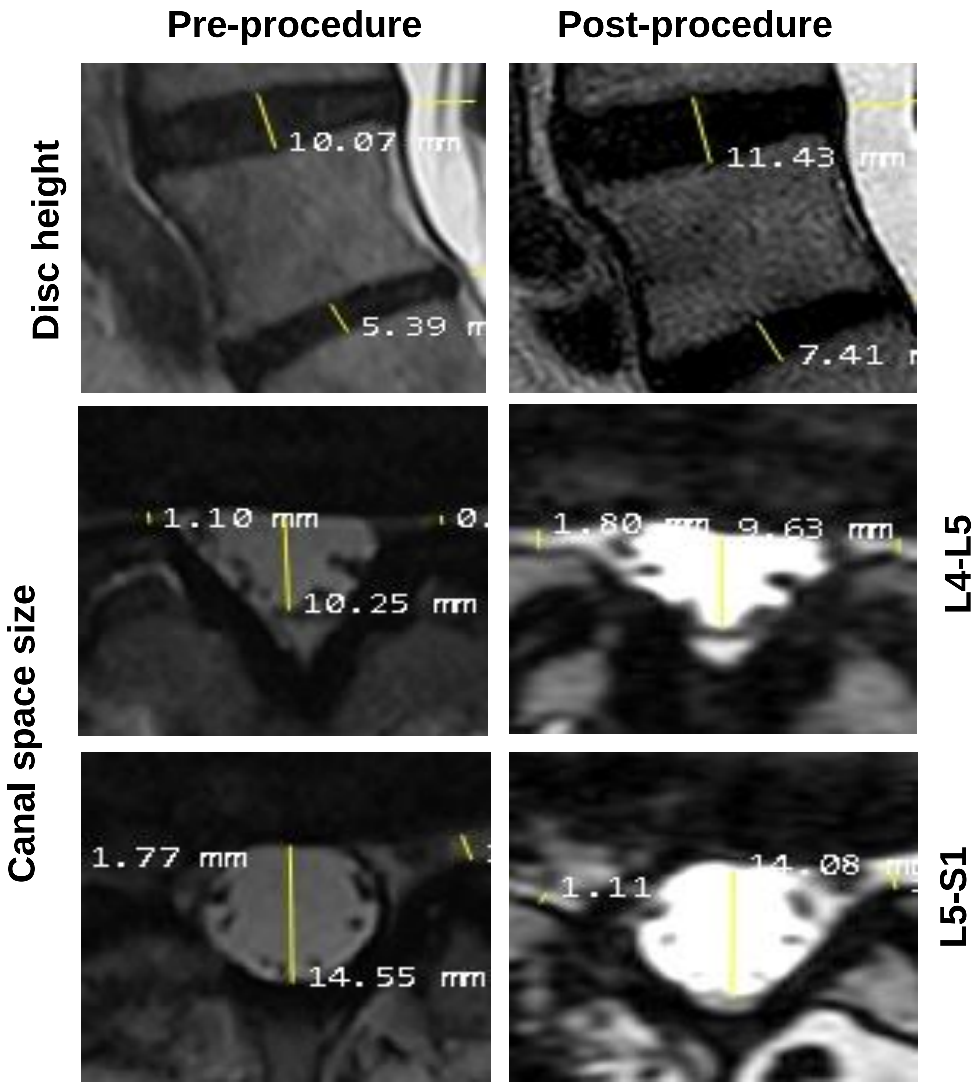

Supplementary Figure S3. MRI pictogram showing the evolution of disc height and canal space size of Patient 5.

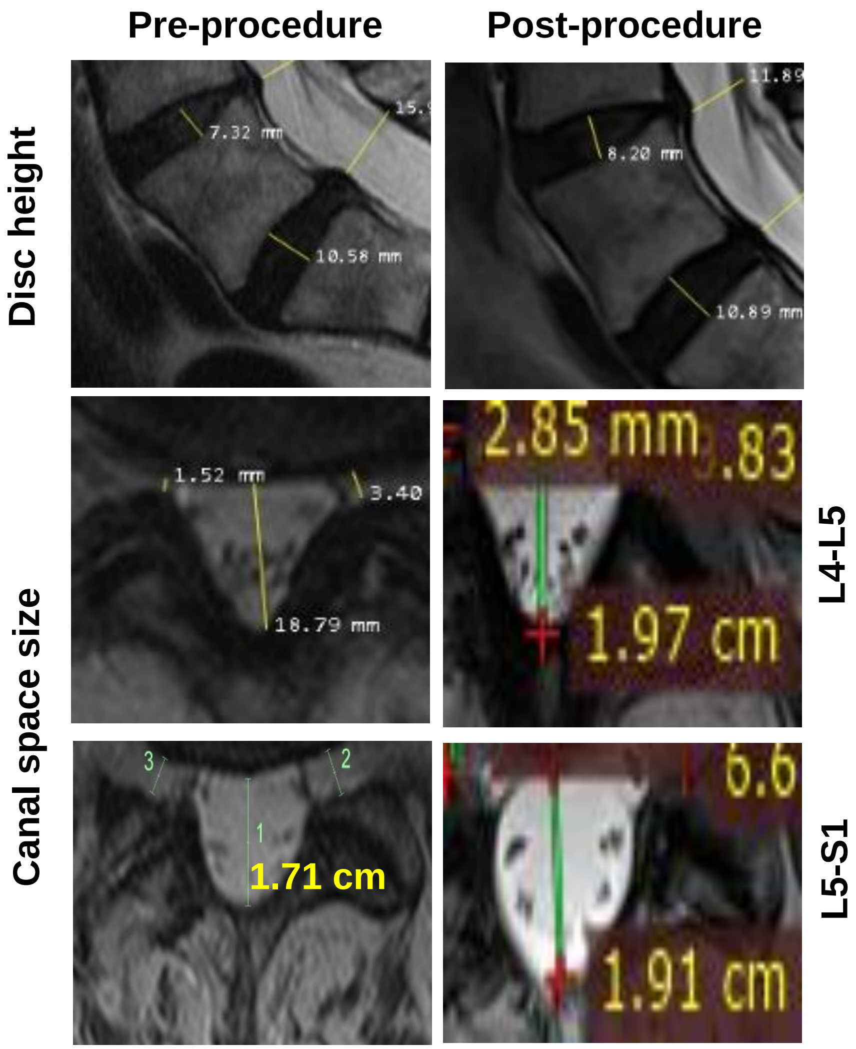

Supplementary Figure S4. MRI pictogram showing the evolution of disc height and canal space size of Patient 9.

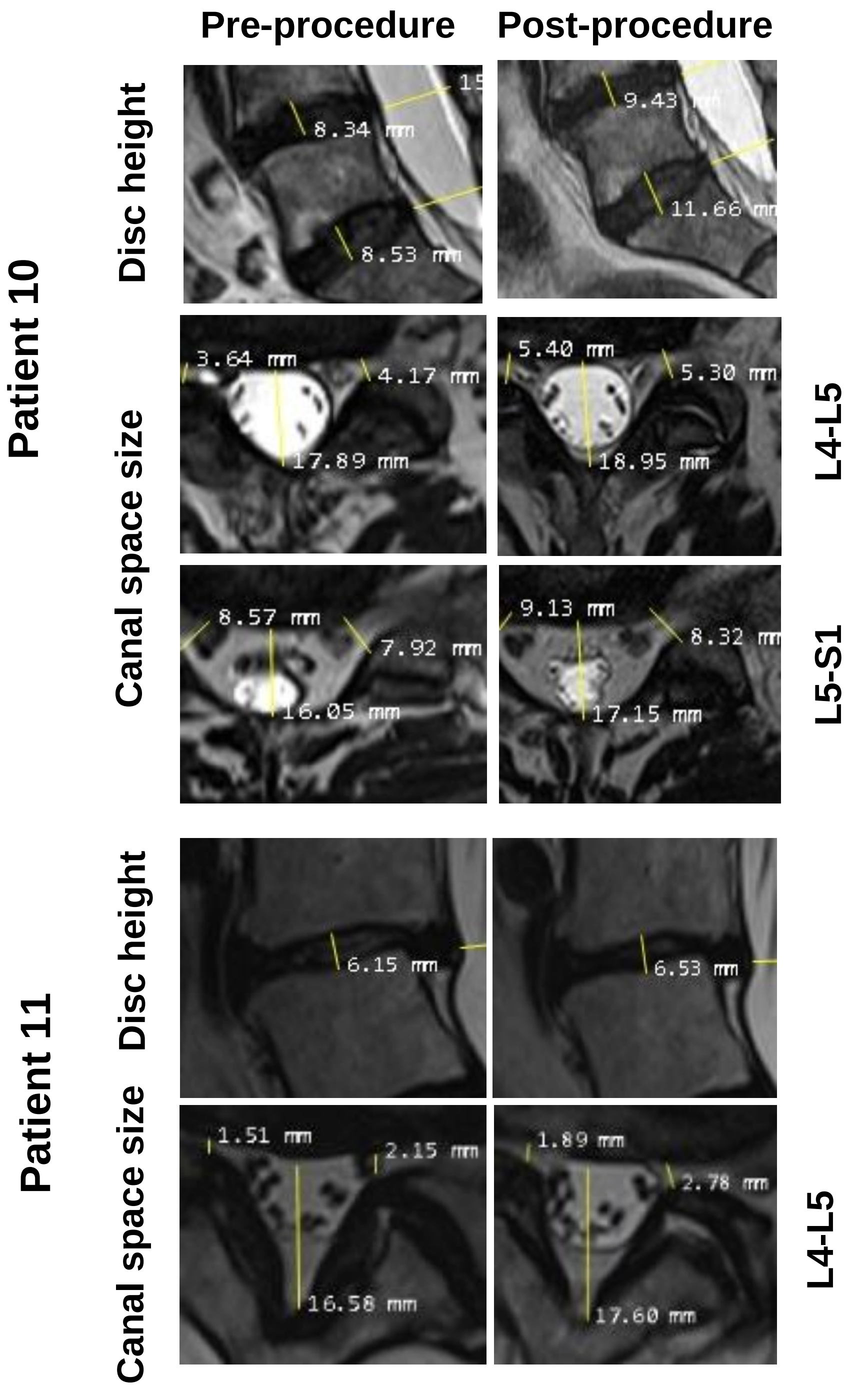

Supplementary Figure S5. MRI pictogram showing the evolution of disc height and canal space size of Patient 10 and Patient 11.

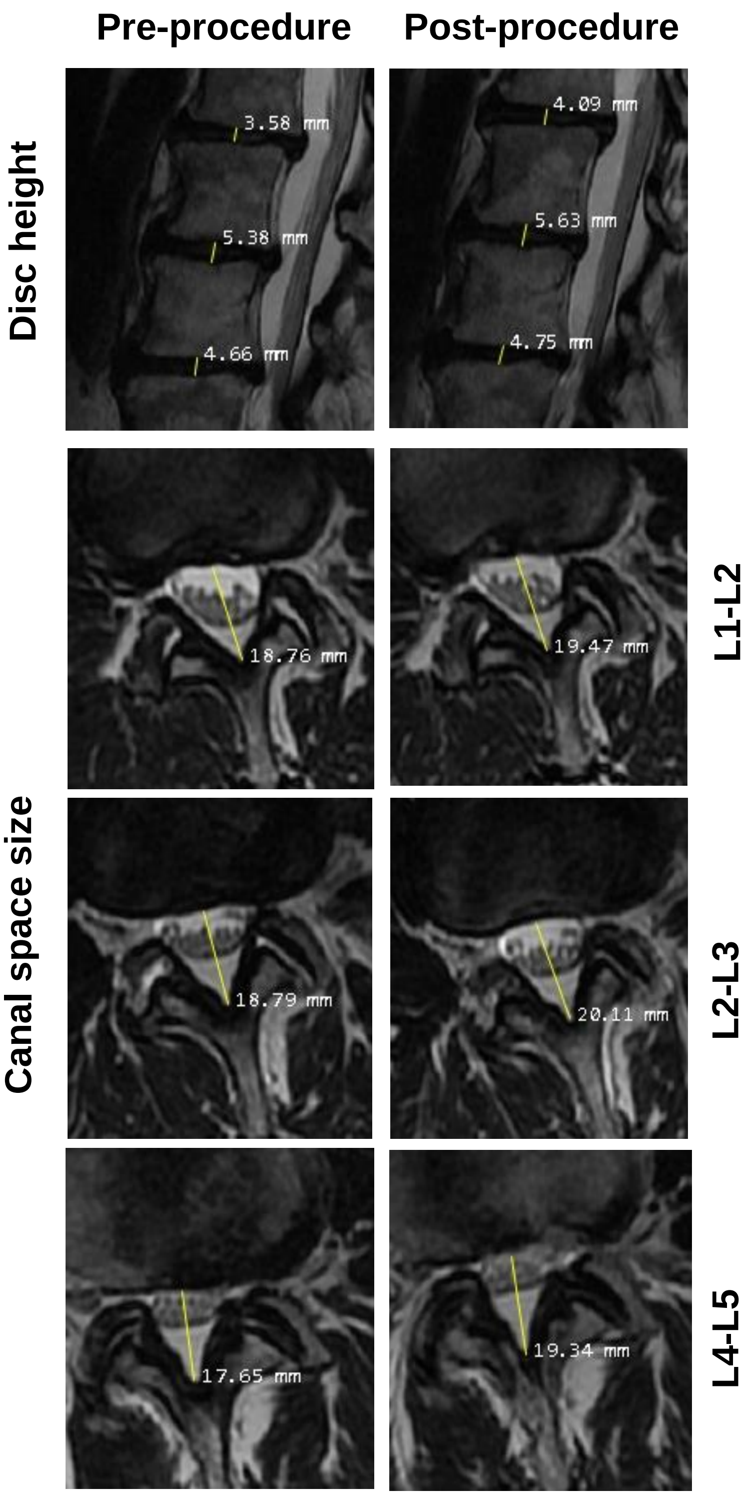

Supplementary Figure S6. MRI pictogram showing the evolution of disc height and canal space size of Patient 13.
